# The emerging roles of aberrant alternative splicing in glioma

**DOI:** 10.1038/s41420-025-02323-0

**Published:** 2025-02-06

**Authors:** Reda Ben Mrid, Sara El Guendouzi, Marco Mineo, Rachid El Fatimy

**Affiliations:** 1 Institute of Biological Sciences (ISSB), Faculty of Medical Sciences, Mohammed VI Polytechnic University (FMS-UM6P), Ben-Guerir, Morocco; 2 Harvey W. Cushing Neuro-oncology Laboratories, Department of Neurosurgery, Harvard Medical School and Brigham and Women’s Hospital, Boston, MA 02115 USA

**Keywords:** CNS cancer, Tumour heterogeneity

## Abstract

Gliomas represent a heterogeneous group of uniformly fatal brain tumors. Low and high-grade gliomas have diverse molecular signatures. Despite successful advances in understanding glioma, several genetic, epigenetic, and post-transcriptional alterations leave various targeted therapies ineffective, leading to a poor prognosis for high-grade glioma. Recent advances have revealed the implication of dysregulated alternative splicing (AS) events in glioma development. AS is a process that produces, from a single genomic sequence, several mature messenger RNAs. Splicing of pre-messenger RNAs concerns at least 95% of transcripts and constitutes an important mechanism in gene expression regulation. Dysregulation of this process, through variations in spliceosome components, aberrant splicing factors and RNA-binding protein activity, disproportionate regulation of non-coding RNAs, and abnormal mRNA methylation, can contribute to the disruption of AS. Such disruptions are usually associated with the development of several cancers, including glioma. Consequently, AS constitutes a key regulatory mechanism that could serve as a target for future therapies. In this review, we explore how AS events, spliceosome components, and their regulatory mechanisms play a critical role in glioma development, highlighting their potential as targets for innovative therapeutic strategies against this challenging cancer.

## Facts

- Glioma is considered to be the most frequent primary form of adult brain cancer.
- AS is a post-transcriptional regulatory process that can produce numerous mRNA from a single gene, resulting in a variety of different protein isoforms.
- Dysregulated AS events have been presently recognized as crucial contributors to tumor development and therapeutic resistance.
- AS aberrations are implicated in glioma proliferation, invasion, and resistance.

## Questions

- Could targeting AS aberrations be an efficient therapeutic method to treat glioma?
- Should AS-related therapeutic methods prioritize individualized stage-specific biomarker-driven treatments, or should they embrace combination therapies that use biomarkers relevant to multiple stages of tumorigenesis?
- Could modifications in AS explain intra-tumoral heterogeneity in Glioblastoma?
- Which strategy has the most potential for rectifying AS changes and, ultimately, improving the therapy of diseases associated with these dysregulations?

## Introduction

Representing 28% of all brain tumors [1], glioma is considered to be the most frequent primary form of adult brain cancer. Deriving from glial stem-like cells in the central nervous system (CNS) [2], adult-type diffuse glioma is characterized by great levels of molecular heterogeneity along with complex genetic alterations [1].

The early molecular profile of glioma describing aberrant Epithelial Growth Factor Receptor (EGFR) transcripts [3] and mutations on the Tumor protein 53 (TP53) tumor suppressor gene [4] established new approaches to cancer characterization, other than histology and immunohistochemistry [5]. The ongoing progress of these investigations has led to the discovery of diverse onco-targets, leading to the emergence of numerous treatment approaches [1].Despite these therapeutic advances, their efficiency is dubious for patients with high-grade glioma (CNS WHO grades 3–4), whose prognosis remains poor despite surgery, radiation, or chemotherapy, with a median overall survival of no more than 20.5 months [6].

Aligning the inspection of diverse molecular features, including genetic biomarkers, telomerase changes, and methylation signatures, has unraveled relatively complex mechanisms of the genesis, growth, and glioma invasion. Along with genetics and epigenetics, AS, an emerging study area in cancer [7], carries promise in understanding partially glioma signaling pathways.

Through the post-transcriptional regulatory process of AS, numerous transcripts are assembled from a single gene, resulting in a variety of different protein isoforms with distinct structures and functions [8]. Dysregulated AS events have been observed in various malignancies [9, 10], and are presently recognized as crucial contributors to tumor development and therapeutic resistance.

Considering the growing association between AS dysregulation and cancer development, this review aims to provide insights on several key aspects of AS’ imbalance in glioma, by focusing on the main features that governs AS patterns, in an attempt to present AS control as a therapeutic option. Extensive alterations in AS have been observed regarding various types of cancers, but the functionality of those changes is often unstudied. The main aim of this review was to collect and critically evaluate studies on AS, with a particular focus on those that contained functional investigations, specifying the exact subtype of glioma where possible or using the broader term “glioma” when the information was not provided.

## Tumoral AS heterogeneity in IDH-WT GBM and IDH-mut gliomas

Molecular heterogeneity is a defining feature of glioma. This heterogeneity constitutes a major challenge and complicates treatment. According to the 2021 WHO classification, tumor heterogeneity of adult glioma has been grouped into 3 subtypes: Isocitrate dehydrogenase-mutant (IDH-mut) astrocytoma (with no 1p19q co-deletion), IDH-mut oligodendroglioma (with 1p/19q co-deletion), and IDH-WT glioblastoma (GBM) [11]. Considerable effort has been made to investigate the aberrant AS in adult glioma, focusing on identifying AS-related signature for subtyping or prognosis purposes [12–14]. In the study conducted by Song et al. [15], most of the AS landscapes between IDH-mut and IDH-WT gliomas distinctly separated from each other. In fact, based on an AS score developed based on the Percent Spliced In (PSI) of top AS events, this score was relatively lower across all cell states in IDH-mut tumors compared to IDH-WT tumors.

While the differences in AS between IDH-mut and IDH-WT subtypes do not necessarily imply that these differences are directly caused by mutational profile, they can offer interesting insights for classifying glioma subtypes and improving diagnosis and management of this cancer. For instance, in the case of MBD1, a member of the methyl CpG-binding domain (MBD) protein family implicated in transcription regulation, Zhang et al. [16] found that the splicing index of exon 10 was increased in the IDH-mutant glioma compared to IDH-WT. This finding suggests a higher expression of an MBD1 isoform containing exon 10 in IDH-mutant gliomas. Another example concerns zinc finger proteins (ZNFs), containing ZNF domains, and which contribute to wide range of biological processes, and which were also reported to be implicated in cancer progression [12]. AS of ZNFs was reported to be associated with IDH status. Indeed, more deletions of ZNF domains in ZNF283, ZNF724P, ZSCAN20, ZNF606, ZNF169, ZNF430, and ZNF20, were observed in IDH-mut gliomas. However, as this phenomenon is not universal in all ZNFs, further studies are required to understand the implication of AS of these ZNFs in glioma.

PTBP1 (polypyrimidine tract-binding protein 1) is known to be a key RNA-binding protein (RBP) in regulating AS under normal and pathological conditions such as in glioma. Recently, Yang et al. [17] showed an upregulation of PTBP1 expression in IDH-WT GBM compared with IDH-mut gliomas. The study further revealed that PTBP1 was essential for the survival of IDH-WT GBM cells by the splicing of CDC42-N, a neuron-specific cell division cycle 42. CDC42 is a small Rho GTPase known to regulate various cellular processes, including actin cytoskeleton remodeling. In IDH-WT GBM, the repression of CDC42-N splicing by PTBP1 ensures the maintenance of actin dynamics required for tumor progression and cell survival [17].

## Emerging trends of alternative splicing dysregulation in glioma

Although extensive profiling of alternative splicing (AS) changes has revealed numerous variations associated with different types of cancers, only a few studies have been able to fully explore the biological implications of these splicing abnormalities. In this review, we will focus mainly on these key studies.

**Fig. 1:**
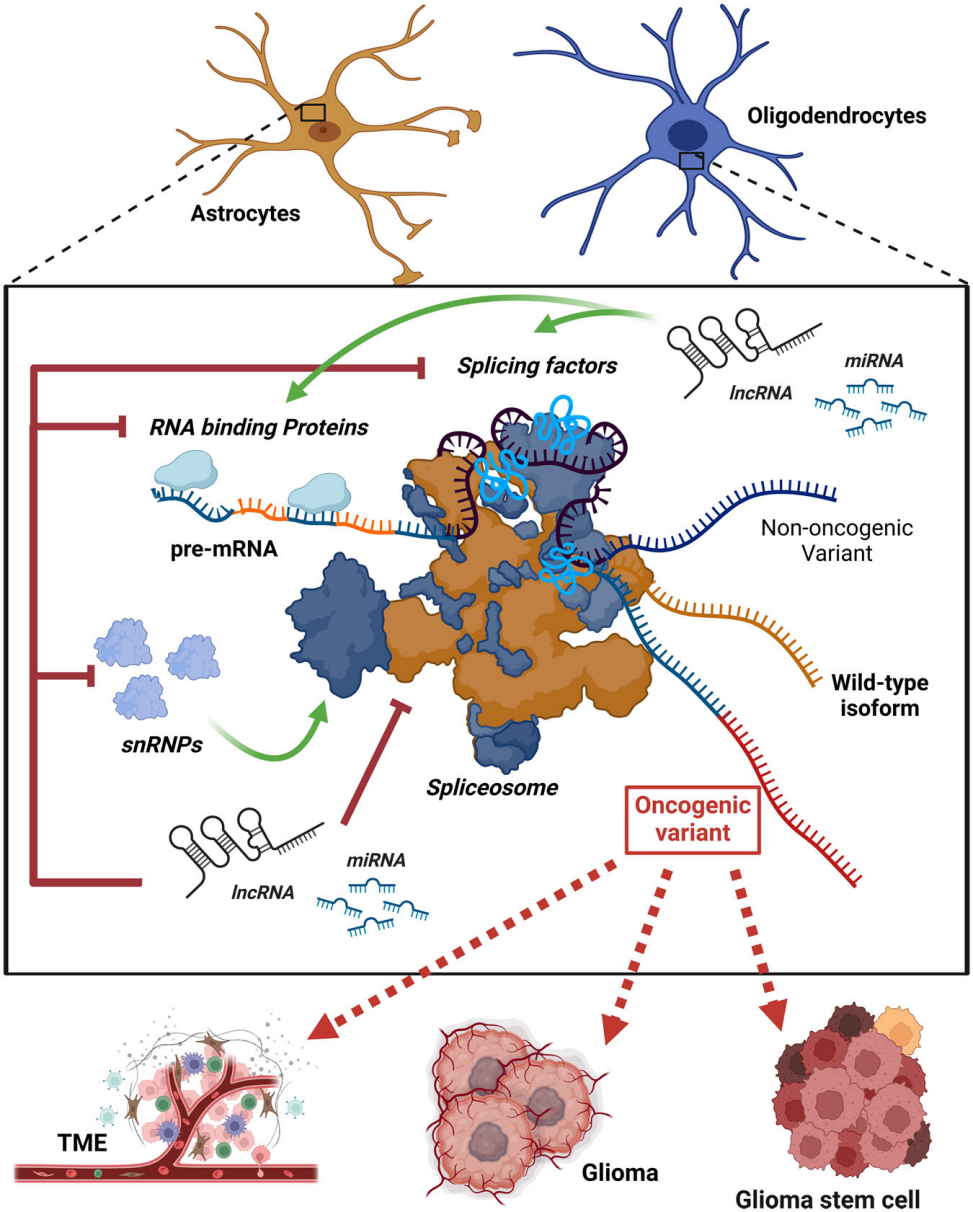
Disruption of the splicing machinery and its regulatory network. This figure illustrates the intricate processes involved in RNA splicing regulation and how its dysregulation contributes to glioma development. At the core of the figure is the spliceosome, a complex molecular machine responsible for the precise splicing of pre-mRNA to produce mature mRNA variants [18]. RNA-binding proteins (RBPs) and splicing factors play pivotal roles in guiding the spliceosome to ensure correct splicing decisions. These proteins influence whether a pre-mRNA is spliced into a wild-type isoform (non-oncogenic variant) or an oncogenic variant. The figure also highlights the involvement of small nuclear ribonucleoproteins (snRNPs), essential components of the spliceosome, in this process. Additionally, non-coding RNAs, including long non-coding RNAs (lncRNAs) and microRNAs (miRNAs), are shown as key background regulators. These non-coding RNAs can modulate splicing either by directly interacting with the spliceosome or by influencing splicing factors and RBPs, thereby promoting or inhibiting the production of oncogenic variants [93]. For instance, oncogene splice variants cause glioma by significantly increasing the rate of progression in tumors and maintaining their stem cell like nature, which is one of their most defining traits. Overall, the figure encapsulates the multifaceted regulation of splicing and its critical impact on glioma biology. “Created in BioRender. El Guendouzi, S. (2025) https://BioRender.com/d48i989”.

Occurring within the nucleus, AS is a process performed by the spliceosome, which also mediates constitutive splicing. The spliceosome is a ribonucleoprotein enzyme machinery composed of multiple small nuclear ribonucleoprotein (snRNPs), that are fundamental for recognizing splice sites and facilitating the precise processing of pre-mRNA [18]. Recent research has shown that the spliceosome is altered in tumors, resulting in oncogenic splicing events that drive tumor development and aggressiveness (Fig. 1). Multiple evidences suggest that AS plays a role in the generation or use of different isoforms of the same gene: a mechanism known as “Isoform switching” [19], which has been hypothesized to contribute to cancer-like features.

One gene of particular interest is *ITSN1* (intersectin 1), which undergoes differential splicing and is currently being investigated for its role in glioma genesis. ITSN1 is a highly conserved protein with multiple domains and two main isoforms: ITSN1-l and ITSN1-s [20]. ITSN1 can interact with proline-rich domain-containing proteins, forming complexes with proteins such as dynamin and SNAP-23/25. These interactions regulate cellular signaling pathways essential for vesicular trafficking, suggesting a critical role of ITSN1 in glioma progression [21]. Subsequent studies have further confirmed the implication of ITSN1 in both endocytosis and exocytosis processes, as well as in regulating various intracellular signaling pathways [22, 23]. A previous study has shown that ITSN1 has distinct cellular distributions for its two isoforms within the central nervous system. ITSN1-l is predominantly found in neurons, while ITSN1-s is ubiquitously detected in microglia and astrocytes [24]. These findings suggest that the unique cellular distribution of these two isoforms corresponds to their functions [25].

ITSN1 has been reported to be involved in cancer cells survival and migration. In fact, Ma et al. [21] showed that siRNA-mediated downregulation of ITSN1-s induced apoptosis of glioma cells through increasing cytochrome c efflux from mitochondria and decreasing anti-apoptotic proteins expression. While ITSN1-s was positively correlated with malignancy of glioma, the ITSN1-l exerted an opposite influence. As per the findings of Shao et al.’s [26], ITSN1-l may be able to reduce the aggressiveness of glioma cells through an inhibitory effect of cell invasion and migration without affecting cell proliferation. In a more recent study, Lan et al. reported that the AS regulating the ratio of ITSN1-s/ITSN1-l was modulated by the activity of PTBP1 [27]. Indeed, this RNA-binding protein (RBP) targeted a sequence on the exon 30 of ITSN1, promoting its inclusion and leading thus to an increased ratio of ITSN1-s/ITSN1-l which further promotes the proliferation and motility of glioma [27].

Dysregulation of AS has also been demonstrated within the glial fibrillary acidic protein (GFAP), which is an astrocytic cytoskeleton type-III intermediate filament protein [28]. Recently, GFAP has gained significant interest due to its implication in multiple physiological processes and diseases. Indeed, it has been reported that the concentration of GFAP in serum could be a possible biomarker for several brain diseases [29]. Ten isoforms have been identified as a result of the AS of the GFAP gene. Different research reports have stated a correlation between glioma grade and the ratio between the splice variants GFAPα and GFAPδ [30]. Although GFAP*α* and GFAP*δ* are the most studied variants, this does not exclude the possibility that the other variants may also serve as excellent biomarkers for gliomas or even serve as more specific biomarkers for each grade of gliomas. This hypothesis is somewhat supported by the newly identified GFAPμ variant, which has been reported to have distinct expression patterns in different glioma subtypes [31].

In addition to their role as biomarkers, some studies have tried to decipher the likely mechanisms that could be responsible for the implication of AS variant GFAP*δ* in the malignancy of glioma. In fact, the study led by Stassen et al. [30], was conducted to investigate the role of GFAP isoforms in astrocytoma malignancy. The authors used data collected from TCGA database and U251-MG astrocytoma cell models to show that a higher GFAPδ/GFAPα ratio, primarily due to reduced GFAPα, induces molecular alterations that regulate tumor-associated genes (e.g., VAV3, NOS2, DUSP4) linked to proliferation and invasion. These changes alter the cell’s interaction with the extracellular matrix, enhancing its invasive capacity, a key feature of high-grade gliomas. The high GFAP *δ*/*α* ratio in grade IV compared to lower grade glioma and its implication in promoting invasiveness have been confirmed in the study conducted by Van Bodegraven et al. [32]. However, additional research is needed to assess the usefulness of the different variants of GFAP both as diagnostic biomarkers and for therapeutic purposes.

Very recently, a study aimed at providing comprehensive AS profiling in glioma by integrating several datasets and validating differential splicing patterns in glioma stem cells (GSCs), clinical samples, and iPSC-based glioma models. The study identified two important isoform switching events related to ceramide synthase 5 (CERS5) and Myelin protein zero-like protein 1 (MPZL1) genes. For CERS5, the study demonstrated that the AS of exon 10, regulated by PTBP1, generated a GBM-associated isoform (iso2), which promotes increased C16-ceramide synthesis in GSCs, enhances GSCs proliferation in vitro, and increases tumorigenicity in vivo. In contrast, iso1, which includes exon 10, is preferentially expressed in normal brain [15]. Regarding MPZL1, two isoforms were reported, E5^+^ include exon 5 and E5^-^ which lack this exon. The E5^+^ isoform, but not E5^-^, was associated with the GSCs proliferation through its interaction with SHP2 and activation of AKT/ERK signaling pathway [15].

## The key regulatory roles of splicing factors in glioma

Splicing factors (SFs) are a class of RBPs that are essential for AS. By interacting with spliceosome and attaching to particular RNA sequences, they control the splicing process [33]. Dysregulation of SFs can lead to abnormal AS patterns, which may result in changes such as exon skipping or inclusion, alternative splice sites, intron retention, or mutually exclusive exons. In addition to affecting protein diversity and mRNA stability, these aberrant splicing processes can aid in the development of tumors [34]. Mutations impacting SFs can also contribute to splicing modifications observed in cancer.

Two major families of SFs have been widely studied: heterogeneous nuclear ribonucleoproteins (hnRNPs) and serine/arginine-rich (SR) proteins. Most SR proteins act as splicing activators due to their ability to bind to exonic splicing enhancers (ESEs) on pre-mRNA and facilitate exon recognition by the spliceosome, thereby promoting exon inclusion. In contrast, hnRNPs generally function as splicing repressors, facilitating exon skipping by binding to exonic or intronic splicing silencers (ESSs or ISSs) [35]. Extensive studies have been conducted on various SFs, shedding light on the prominent role of these RBPs in glioma genesis [36].

### SR protein family

**Fig. 2:**
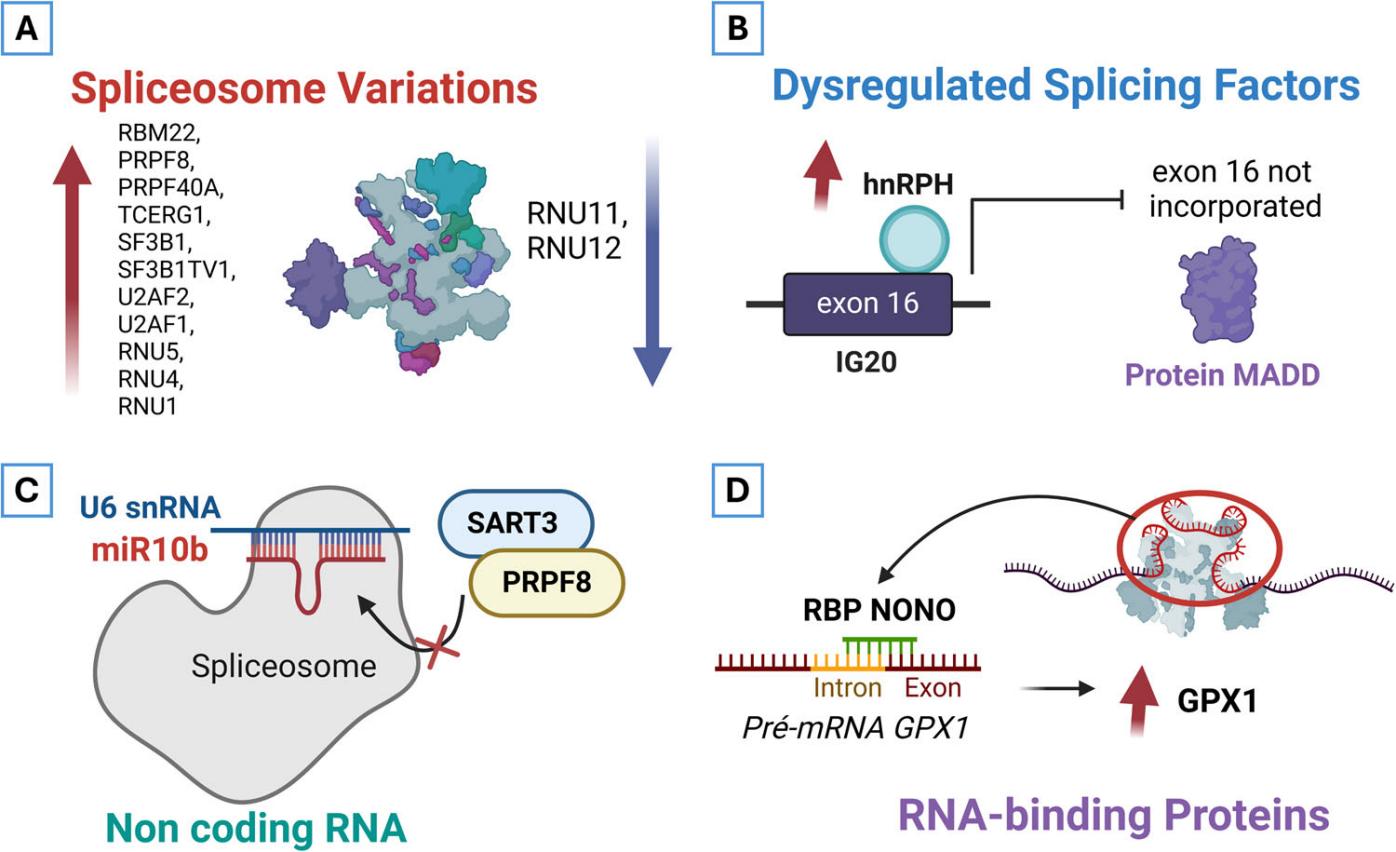
Dysregulated alternative splicing events in glioma. **A** Fuentes-Fayos et al. [37] uncovered the upregulation within GBM of 11 specific components of spliceosome-associated transcripts (RBM22, PRPF8, PRPF40A, TCERG1, SF3B1, SF3B1TV1, U2AF2, U2AF1, RNU5, RNU4, and RNU1), as well as the downregulation of 2 others (RNU11 and RNU12). These findings provide evidence for the implication of AS in glioma [37]. **B** The splicing of IG20 is regulated by the SF hnRNPH, which is elevated in gliomas [46]. By attaching to the UGGG-containing silencer at the 5’ end of exon 16, hnRNPH prevents exon 16 from being incorporated, which produces MADD, an antagonistic, anti-apoptotic isoform of IG20 [50]. This isoform reroutes TNF-a/TRAIL-induced death signaling, encouraging cell growth. **C** (1): El Fatimy et al. have provided evidence that miR-10b, specifically elevated in malignant gliomas, controls U6 snRNA levels, stability, conformation, and levels of N-6-adenosine methylation and pseudouridylation, as well as U6 binding to SF SART3 and PRPF8. These effects on U6 are exemplified by decreased levels of GTPase CDC42, and consequences on cell viability mediated by CDC42 [54]. **D** According to X. Wang, Han et al., the RBP NONO interacts with GPX1 pre-mRNA and encourages its splicing binding to its promoter, which increases the expression of GPX1 mRNA. In GBM cells, this increase of GPX1 improves antioxidant capacity and decreases oxidative stress, encouraging cell proliferation, migration, and invasion. A recent study found that the ubiquitination of NONO by TRIM25 protein, promote tumor invasion. Defective, NONO retained the second intron in PRMT1 pre-mRNA, limiting PRMT1/c-MYC pathway activation [80].“Created in BioRender. El Guendouzi, S. (2025) https://BioRender.com/g77p377”.

In 2020, Fuentes-Fayos et al. found significant disruptions in the expression of spliceosome components and splicing factors in high-grade gliomas, compared to healthy brain tissue, particularly in GBM (Fig. 2). They identified four splicing factors (SRSF3, RBM22, PTBP1, and RBM3) that could accurately distinguish between tumor samples and control samples, as well as different subtypes of tumors from mouse models with gliomas. These findings were confirmed in multiple independent human cohorts [37]. Silencing these splicing factors reduced the aggressive behavior of U-87 MG and U-118 MG tumor cells and induced apoptosis, particularly when SRSF3 was silenced in vitro. In in vivo experiments, silencing SRSF3 significantly decreased tumor development and progression, likely by affecting the platelet-derived growth factor receptor beta (PDGFRB) pathway, which plays a critical role in tumor growth and angiogenesis [37].

The study of other splicing factors implicated in aberrant splicing indicated that SRSF10, which is upregulated in glioma-associated endothelial cells [38], contributes to this process by promoting the biogenesis of circular-ataxin 1 (circ-ATXN1) biogenesis. Notably, ATXN1 was reported to be implicated in the development of neurodegenerative diseases [39]. In glioma-associated endothelial cells (GECs), MiR-526b-3p expression is downregulated and has antitumor effects. By binding to miR-526b-3p, Circ-ATXN1 functionally targets miR-526b-3p and promotes GECs angiogenesis [39]. Liu et al. [38] investigated glioma angiogenesis using glioma endothelial cells and identified the SRSF10/circ-ATXN1/miR-526b-3p axis as a critical regulatory pathway. The knockdown of SRSF10 and circ-ATXN1 significantly reduced GEC proliferation, migration, and tube formation in vitro and reduced glioma angiogenesis in vivo. The observed effects were further enhanced by the overexpression of miR-526b-3p, which exerted anti-angiogenic effects by targeting MMP2 and VEGFA.

Another example of aberrant expression of SFs in glioma concerns the overexpression of the splicing activator TRA2A (Transformer-2 Alpha) in high-grade gliomas (III and IV) and GBM cell lines [40]. TRA2A was reported to increase the expression of the cell proliferation marker Ki67, thereby promoting the proliferation of U251 GBM cell line. It also increased vimentin and neural cadherin (N-cadherin) while decreasing epithelial cadherin (E-cadherin), thereby promoting the epithelial-to-mesenchymal-like transition (EMT-like). These changes in protein expression speed up the proliferation, migration, invasion, and EMT-like of glioma cells [40].

### hnRNP protein family

Different hnRNPs have been reported to regulate AS [41]. In GBM, Golan-Gerstl et al. [42] discovered that the overexpression of the splicing factor hnRNPA2/B1 results in the expression of oncogenic forms of tumor suppressors like BIN1 and WWOX. This also affects the expression of anti-apoptotic proteins encoded by casp8 and fadd-like apoptosis regulator (CFLAR) and caspase 9 (CASP9), the insulin receptor (INSR), and the proto-oncogene MST1R. These results suggest that hnRNPA2/B1 is a potential proto-oncogene [42]. Inhibiting hnRNPA2/B1 causes the cell cycle to be interrupted in the S phase and reduces the phosphorylation of the AKT and STAT3 signaling pathways in U251 glioma cells [43]. Furthermore, knockdown of hnRNPA2/B1 leads to reduced GBM cell P-STAT3 and MMP-2 activation as well as decreased cellular migration and invasion [44]. In another study [45], hnRNPA2/B1 was found to form a complex with the RNA-binding protein SON (Ser/Arg (SR)-related protein), playing a crucial role in regulating RNA splicing and the switch between PTBP1 and PTBP2 in GBM. SON is notably overexpressed in GBM, where it contributes to the upregulation of PTBP1 by promoting intron removal. Simultaneously, SON suppresses the expression of PTBP2, a protein involved in neuronal differentiation, by interacting with hnRNPA2/B1 and modulating AS [45].

HNRNPH1, another member of hnRNPs family, is overexpressed in GBM and has been shown to control the AS of oncogenic isoforms. Through the same mechanism used in the modulation of IG20/MADD exon 16 [46] (Fig. 1), HNRNPH1 controls RON exon 11 splicing. The knockdown of HNRNPH1 reduces the invading capability of GBM cells to migrate, showing that HNRNPH1 levels contribute to invasion properties of GBM, through modulation of RON and IG20 oncogenic splicing [46]. Notably, it has been shown that HNRNPH1 binds the mRNA of several genes with immune-related functions, suggesting that its implication in modulating AS may also play a central role in shaping the immunosuppressive GBM microenvironment [47]. In a similar line, GBM exhibits a substantial elevation in PTBP1 expression levels (also known as hnRNPI) [48]. As previously mentioned, PTBP1 regulates the AS of multiple target genes, among these are fibroblast growth factor receptor-1 (FGFR-1), pyruvate kinase M (PKM), and multidrug resistance protein 1 (MRP1) [49], contributing to the drug-resistant phenotype frequently associated with GBM [50].

Several other reports have highlighted the implication of SFs dysregulation and subsequent aberrant AS in glioma development, which may result in increased cell proliferation, migration, invasion, and the development of malignant features (Table 1).

**Table 1:** Dysregulated splicing factors, hnRNPs, and RNA-binding proteins involved in aberrant alternative splicing in glioma.

	SF or RBP	Expression in glioma	Aberrant AS mechanism	Target	Result	Cellular process	Ref.
PTBP1	SF	Overexpression	Repressed splicing	CDC42-N: Small Rho GTPase	Isoform maintaining actin dynamics	Tumor progression and cell survival	[17]
			Inclusion of exon 30	ITSN1: Regulate vesicular trafficking,	Increased ratio of ITSN1-s/ITSN1-l	Proliferation and motility of glioma cells	[27]
			Exclusion of exon 10	CERS5: Synthesis of ceramide	Increased C16-ceramide synthesis by Iso2	Proliferation and tumorigenicity	[15]
hnRNPA2/B1	hnRNP	Knockdown	--------------	Suppressing AKT and STAT3 phosphorylation	Inhibition of the expression of PCNA, CyclinD1, and Bcl2	Limiting proliferation and increase of apoptosis	[43]
			--------------	STAT3	Reduced activation	Limiting cell proliferation, migration, and invasion	[44]
hnRNPH	hnRNP	Overexpression	Exon Skipping: Exon 11	RON: Tyrosine kinase receptor	RON exhibits a truncation in its extracellular domain.	Invasiveness and motility of tumor cells	[46]
hnRNPA2/B1	hnRNP	Overexpression	Skipping of exons 6 to 8	WWOX: Tumor suppressor	Deletion of its substrate binding domain and its alcohol dehydrogenase domain	Inactivation of its anti-invasive and antiapoptotic functions.	[42]
			Exon Skipping: Exon 11	INSR: member of the receptor tyrosine kinase	Generates the mitogenic isoform/splicing variant IR-A : that binds the growth factor insulin-like growth factor II (IGF-II)	Implication in an autocrine loop in cancer cells.	
			--------------	CFLAR: an antiapoptotic protein	Increased level of the long isoform	Inhibition of TNF and TRAIL-induced apoptosis; enhance motility and invasion through activation of the MAPK-ERK pathway	
			Skipping of exons 3 to 6	CASP9: gene coding for caspase-9	Truncated dominant negative anti-apoptotic isoform caspase-9B	Inhibition of apoptosis	
			Inclusion of exon 12a	BIN1: Tumor suppressor	Inactivation of its tumor suppressor activity	Generation of antiapoptotic isoform	
SRSF10	SF	Upregulation	Binds to the 3’ and the 5’ ends of circ-ATXN1 pre-mRNA	circ-ATXN1	Stimulating circ-ATXN1 biogenesis: Circ-ATXN1 binds to miR-526b-3p and inhibits its expression *(miR-526b-3p prevents GEC angiogenesis)*.	Angiogenesis, Glioma growth,	[38]
TRA2A	SF	Upregulation	Controls a splicing switch among alternative and constitutive target exons	Ki67, Vimentin, N-cadherin	Upregulating Ki67, Vimentin, N-cadherin; Downregulating E-cadherin	Promotion of EMT, proliferation, migration	[40]
HnRNPD	RBP	Upregulation	Binds to the 3’UTR of ZHX2 promoter	ZHX2, linc00707, miR-651-3p, SP2	Controlling both expression and stability of ZHX2 targeted genes	Increasing vasculogenic mimicry in glioma cells	[91]
BAF45d	RBP	Upregulation	Binds to PTBP1 promoter	PPP3CC, EGFR, EZH2, PILRA	Influencing the splicing patterns of PTBP1 target genes	Promotion of glioma growth	[92]

The table presents key SFs, hnRNPs, and RBPs driving aberrant alternative splicing events in glioma. SF, hnRNPs, and RBP dysregulation can result in aberrant splicing patterns such as constitutive splicing, exon skipping or inclusion, alternative splice sites, intron retention, and mutually exclusive exons. These abnormal splicing processes, in addition to altering protein diversity and mRNA stability, can contribute in tumor formation.

## Non-coding RNAs modulating alternative splicing in glioma

Non-coding RNAs (ncRNAs) are involved in many different biological processes, particularly in pathological conditions. The synthesis of splicing isoforms is regulated by ncRNAs, among them micro-RNAs (miRNAs), circular RNAs (circRNAs), long non-coding RNA (lncRNAs), and small nuclear RNA (snRNAs) (Fig. 1). These regulators work by either directly or indirectly affecting molecular targets that regulate pre-mRNA transcription, trans-acting factors, cis-acting elements, or both at different phases of the process. Due to their ability to modify cellular signaling pathways that either stimulate or impede the genesis of cancer, they may prove to be useful targets for therapy or diagnosis.

### microRNA as background regulators of alternative splicing in glioma

Investigating the role of ncRNAs in modulating AS reveals that miRNAs mainly affect the target mRNA’s 3’UTR and occasionally its 5’UTR [51]. As previously mentioned, PTBP1 regulates the AS of multiple target genes. PTBP1 has been reported to be directly targeted by the neuron-specific microRNA-124 (miR-124). The reduction of PTBP1 leads to the accumulation of PTBP2, an important process for the differentiation of progenitor cells to mature neurons [52]. In GBM, a study showed that the loss of miR-124 leads to PTBP1 overexpression, which drives aberrant splicing of a brain-specific exon in the tumor suppressor ANXA7. This splicing enhances EGFR signaling, promoting GBM progression [53].

It has been demonstrated that mir-10b directly binds to U6 snRNA, acting as a key contributor to the splicing of pre-mRNA within the spliceosome molecular machinery. This molecular interaction change conformation, stability, and levels of U6 snRNA, which in turn affects the AS of key genes involved in GBM progression [54]. Moreover, this investigation demonstrated how miR-10b regulates the AS CDC42, a process that is critical for GBM cell growth and survival [54].

MicroRNAs have the ability to indirectly influence the regulation of AS through their function in controlling the activation of particular transcription factors [55]. Indeed, it has been shown that miR-150-3p is downregulated in glioma tissues and cell lines, and its overexpression inhibits glioma cell growth by targeting the transcription factor specificity protein 1 (SP1). SP1 regulates the expression of genes involved in cell division, proliferation, apoptosis, and angiogenesis and it has been reported to be overexpressed in certain cancers, including glioma [56]. Despite the growing interest in miRNA’s function in this complex process, the miRNAs involved in the control of AS in gliomas remain a major area of investigation.

## Long non-coding RNAs in the regulation of alternative splicing in glioma

Long non-coding RNAs are RNA molecules that exceed 200 nucleotides in length yet do not encode proteins. They can interact directly with the splicing machinery, influencing the regulation of AS. LncRNAs have the ability to alter the mRNA cleavage, translational repression, or epigenetic landscape of target genes, affecting gene expression and cell function. The targeted pre-mRNA interacts with lncRNAs in both cis-natural antisense transcript (cis-NAT) and trans-NAT forms [57]. LncRNAs employ various strategies to regulate SFs in cancer. Among the most extensively studied is the lncRNA MALAT1 [58] (Fig. 2). MALAT1 is predominantly located in nuclear speckles, which are dynamic, membraneless subnuclear structures involved in the modification and assembly of splicing factors [59]. It has been reported that MALAT1 regulates AS by stabilizing the interaction between the splicing factors PTB-associated SF (PSF), and PTBP1, forming a functional complex that associates with pre-mRNAs and affects multiple AS events in hepatocellular carcinoma [59]. While MALAT1 has been reported to be overexpressed in gliomas and associated with pathogenesis and chemoresistance [60], the specific molecular mechanism by which MALAT1 influences AS of its targets in gliomas, has yet to be determined.

In GBM, the lncRNA HIF1A-AS2 interacts with the RBPs IGF2BP2 and DHX9 to promote the expression of HMGA1 (high mobility group A1) mRNA [61]. HMGA1 is responsible for modulating the expression of different genes and key cellular functions [62]. Importantly, the degree of malignancy correlates with HMGA1 expression levels, and according to Liu et al. [63], GBM patients with higher HMGA1 levels exhibit significantly shorter progression-free survival times.

## The implication of alternative splicing in glioma resistance

**Fig. 3:**
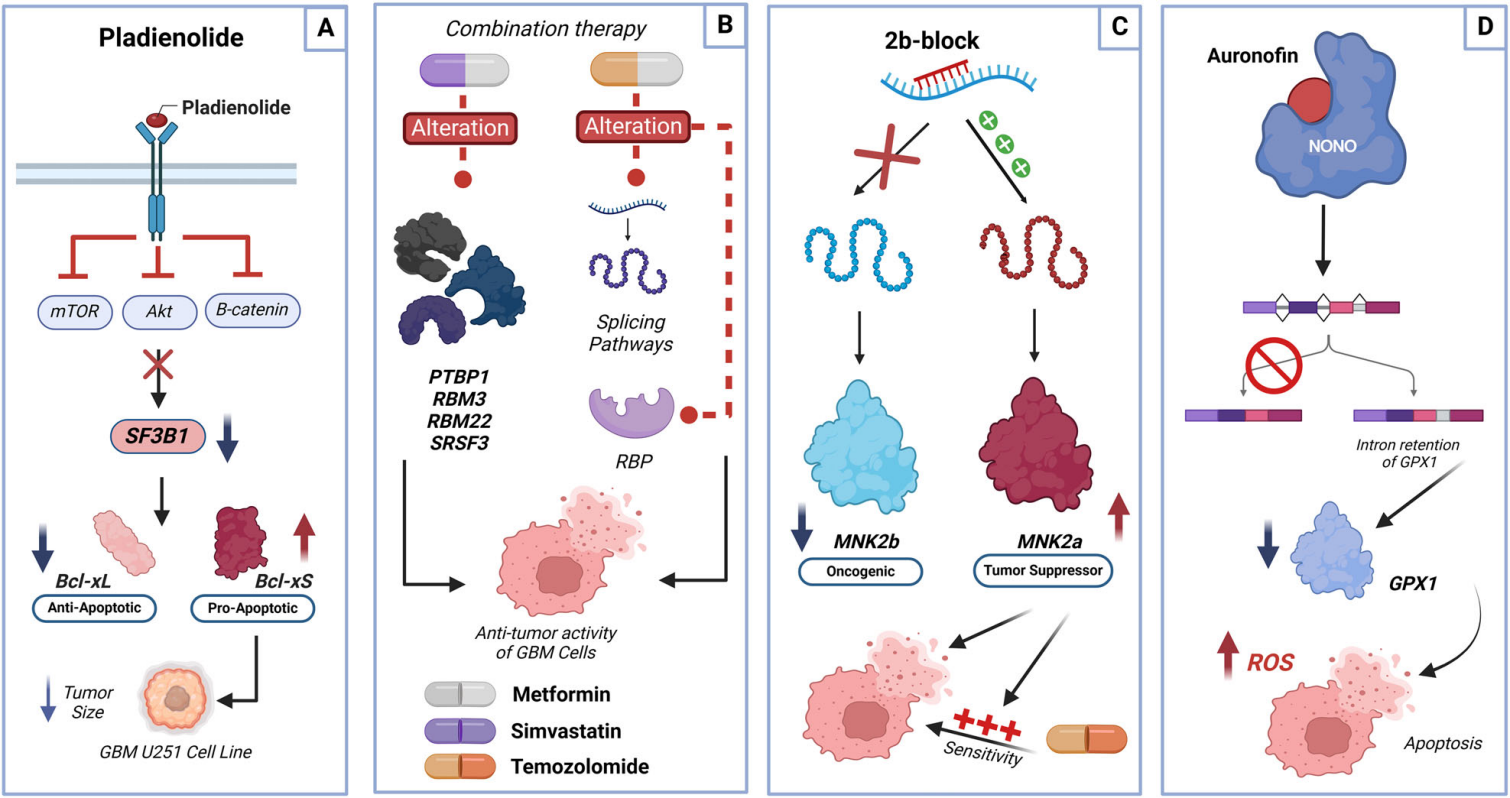
Controlling splicing for therapeutic benefit. **A** Pladienolide B targets SF3B1, implicated in the recognition of 3’ splice site [94], and inhibits its activity both in vitro and in vivo leading to a decrease in cell proliferation, secretion of VEGF, along with an increase in apoptosis. These effects were most likely achieved by suppressing the AKT/mTOR/ß-catenin pathways and misbalancing the splicing of BCL2L1 by upregulating the pro-apoptotic Bcl-xS, while downregulating anti-apoptotic Bcl-xL [95]. **B** Metformin, associated with TMZ, exhibit strong antitumor activity in GBM. Metformin combined to simvastatin showed potent effect against GBM cell compared to individual treatments, hypothesized to be altering the expression of the critical oncogenic genes implicated in the spliceosome machinery (PTBP1, SRSF3, RBM22, and RBM3) [96]. **C** 2b-block is a specific SSO, evaluated for its effectiveness in treating GBM. To manipulate MKNK2 AS and promote the formation of Mnk2a (Tumor Suppressor isoform) [64], evaluated the effect of 2b-block in U87MG cells. 2b-block prevents the splicing machinery from accessing the splice site responsible for Mnk2b (the oncogenic isoform) which favors the upstream splice site, resulting in increased levels of Mnk2a inhibiting survival of GBM cells and sensitized them to chemotherapy [64]. **D** Wang et al. [97] reported that NONO was overexpressed and implicated in the progression of GBM by regulating splicing of GPX1. The inhibition of NONO using a small molecule inhibitor, Auranofin, results in intron retention and impaired thus the expression of GPX1. Decreasing GPX1 leads to apoptosis and blocks the invasion through an increase of reactive oxygen species. Consequently, Auranofin may constitute a candidate to treat GBM [97]. “Created in BioRender. El Guendouzi, S. (2025) https://BioRender.com/w83g233”.

In the case of GBM, AS has been implicated in the acquisition of cellular resistance to chemotherapy, as reported by several studies. One of these studies was carried out by Mogilevsky et al. [64] to investigate the role of the *MKNK2* gene encoding the Mnk2 kinase. This protein is part of the MAPK pathway, which plays an essential role in mRNA translation initiation through the phosphorylation of the eukaryotic translation initiation factor 4E (eIF4E). The phosphorylation of eIF4E by the Mnk2 proteins enhances the export and translation of different mRNAs involved in tumorigenesis, thereby promoting the oncogenic activity of eIF4E. In humans, Mnk2 pre-mRNA can be subjected to AS leading to two isoforms: Mnk2a and Mnk2b. This AS event results in two proteins with different functional characteristics and possible regulatory interactions. In particular, the long isoform named Mnk2a contains a MAPK binding site which is missing in the short isoform Mnk2b (Fig. 3). SRSF1 is one of the SR protein family members responsible for the regulation of the *MKNK2* gene. Overexpression of SRSF1 increases the level of MnK2b and reduces the level of Mnk2a. Downregulation of Mnk2a has been reported in several cancers [64]. In the previous study, splice switching oligonucleotides (SSOs) have been used to investigate the implication of Mnk2 isoforms in GBM progression and drug resistance. The results showed that the induction of Mnk2a by using a specific SSO was responsible for inhibiting the oncogenic properties of GBM and re-sensitized them to chemotherapy [64].

GBM is highly heterogeneous, which presents significant challenges for treatment failure. Four major states of GBM cells have been identified by researchers: neural progenitor-like (NPC), oligodendrocyte progenitor-like (OPC), astrocyte-like (AC), and mesenchymal-like (MES) states [65]. These states are influenced by the tumor microenvironment and oncogenic drivers, which enable GBM cells to develop resistance to treatments through phenotypic switching or plasticity-mediated transformation [66]. Note that the MES subtype is characterized by high invasiveness, poor prognosis, and treatment resistance. It has been demonstrated that the activation of NF-κB could lead to MES transition in GSCs [67]. In a study conducted by Li et al., the researchers examined how splicing regulation contributed to GBM heterogeneity and the occurrence of the MES phenotype. The focus was on SR-related proteins, splicing factors with an arginine/serine (RS) domain that participate in spliceosome assembly. The study was able to identify a new SR-related protein—arginine/serine-rich protein 1(RSRP1)—that was highly expressed in MES GSCs as compared with other subtypes. RSRP1 promoted the MES phenotype through splicing regulation. They established that RSRP1 affects several genes’ splicing patterns including Poly (ADP-ribose) polymerase family member 6 (PARP6), which is a tumor suppressor. Exon skipping induced by RSRP1 on PARP6 resulted in a shorter isoform lacking a catalytic triad, leading to increased NF-κB activation and facilitating MES GBM phenotype expression [68]. Specifically, RSRP1 promotes the skipping of exon 18 in PARP6, resulting in the formation of a pathogenic isoform that lacks the catalytic triad. The authors discovered that spliceosome- and NF-κB-targeted medicines had a synergistic antitumor impact on GBM, implying that targeting RSRP1 function could be a potential treatment for MES GBM [68]. Pladienolide B and BAY11-7082, acting as inhibitors of the spliceosome and NF-κB pathway, demonstrated therapeutic efficacy against MES GBM in this study, as showed by both in vivo and in vitro testing. Because MES transition occurs in individuals with treatment-resistant GBM, this spliceosome and NF-kB-targeting combination method may be beneficial for patients with recurrent illness. Furthermore, patients with GBM characterized by high spliceosome activity, or RSRP1 overexpression may be candidates for such treatments [68].

Radiation has also been reported to induce AS events, which may play a role in the radiation-induced regulation of gene expression [69]. Radiation influences not only the creation of new splicing events but also the selective translation of existing mRNA isoforms, highlighting a potential role in radiation-induced translational control and cellular response to radiation [70]. Apoptosis regulator Bcl-extra (BCLX) is a member of BCL2 family proteins implicated in the regulation of cell fate [71]. BCLX, which is also known as BCL2L or BCL2L1, can generate two alternatively spliced isoforms, the anti-apoptotic Bcl-xL and pro-apoptotic Bcl-xS isoforms. The implication of Bcl-xL in radiation resistance has been already discussed [72]. The study conducted by Dou et al. [73], indicated that combining X-ray radiotherapy with BCLX splicing modulation can play a significant role in radiosensitivity of GBM. In fact, they revealed that targeting the aberrant splicing of Bcl-xL, using specific SSOs (named Bclx-vMO), was able to reduce Bcl-xL, increase Bcl-xS, and enhance the radiosensitivity of GBM cells. As SF3B1 has been shown to be responsible for misbalancing the splicing of BCLX, targeting SF3B1 led also to an upregulation of the pro-apoptotic Bcl-xS, while downregulated the anti-apoptotic Bcl-xL **(**Fig. 3**)**. Therefore, targeting the aberrant splicing of Bcl-xL or the core spliceosomal protein SF3B1 could constitute important therapeutic targets for the treatment of GBM and deal with radioresistance.

Intriguingly, treating GBM by targeting different parts of spliceosomes or abnormally spliced isoforms is an interesting concept. This might reveal new and inventive approaches to disrupting molecular processes that occur during GBM and probably enhance the outcomes of treatment.

## Alternative splicing and m^6^A modification in glioma

The most prevalent form of eukaryotic mRNA methylation is N6-methyladenosine (m^6^A), which regulates diverse aspects of transcriptome function. The enzymes involved in the m^6^A methylation processes are methyltransferases (writers), demethylases (erasers), and binding proteins (readers). Recent studies have elucidated functional roles for m^6^A methylation in mRNA splicing and cancer development. For instance, methyltransferase 3 (METTL3) is a well-known m^6^A writer that promotes mRNA translation and regulates tumor cell proliferation through the methylation of target genes [74]. Insulin-like growth factor 2 mRNA-binding protein 3 (IGF2BP3), a member of the m^6^A-reading family, enhances mRNA stability and translation by recognizing m^6^A-containing sequences [75].

As described previously, SR proteins, a group of splicing regulators, promote exon inclusion and are involved in various diseases [76]. Among the SR proteins family, abnormal expression of SRSF5 has been reported in breast, renal, and lung cancers, and it was involved in AS events in prostate and lung cancers [77]. In a study conducted by Zhang et al. [78], the authors investigated the role of SRSF5 in blood-tumor barrier (BTB) permeability regulation. As it is known, the BTB impedes the delivery of chemotherapeutic drugs to glioma tissue, limiting treatment efficacy. Therefore, the selective permeability of the BTB represents a potential strategy to enhance chemotherapy effectiveness in glioma. The authors showed that METTL3, IGF2BP3, CPEB2, and SRSF5 were upregulated in GECs. The study of the upstream and downstream effects of the upregulation of these genes showed that METTL3 promoted m^6^A methylation of CPEB2 mRNA, while IGF2BP3 enhanced its stability. CPEB2, in turn, stabilized SRSF5 mRNA and regulated its AS activity on ETS1. ETS1 can generate multiple splice variants. SRSF5 was shown to be responsible for ETS1 exon-7 inclusion and formation of P51-ETS1 which stimulates the expression of tight junction-related proteins such as zonula occludens-1 (ZO-1), occludin, and claudin-5, responsible for regulating BTB permeability [78].

## Controlling splicing for therapeutic benefit

Cancer is characterized by dysregulated AS events promoting the generation of diverse RNA isoforms. To this effect, several therapeutic approaches may be proposed, which may target splicing at various levels of its regulation. These could include targeting splicing factors or their regulators, targeting components of the spliceosome machinery, using oligonucleotides to block the synthesis of aberrant isoforms or targeting abnormal protein isoforms as illustrated in Fig. 3.

The x‐box binding protein 1 (XBP1), a transcription factor that is activated after removal of 26‐nucleotide introns, thus converting XBP1 to its spliced active form (XBP1s), has been found to be overexpressed in GBM. XBP1s was reported to be critical in determining cell fate in response to endoplasmic reticulum (ER) stress [79]. Targeting XBP1s using the small molecule inhibitor MKC‐3946, reduced the growth of GBM cells and enhanced the effectiveness of temozolomide (TMZ) in GBM cells [80]. Temozolomide is known to specifically target guanine bases, essential nucleotides in various RNA and DNA secondary structures, including G-rich regions such as RNA splice sites. A recent study revealed that TMZ-resistant GBM is characterized by mutations in guanine bases that disrupt splice sites, leading to deregulated AS [81]. The study also showed that cdc2-like kinases (CLK) family of splicing regulatory kinases, responsible for the hyperphosphorylation of SR proteins, was increased in TMZ-resistant cells to maintain the phosphorylation profile of SR proteins, unlike in TMZ-sensitive cells. A newly identified inhibitor of CLK2 demonstrated potent effects on TMZ-resistant glioma cell lines offering hope for new therapies targeting TMZ-resistant GBM [81].

The enigmatic potential of RBPs and miRNAs across the spliceosome remains largely unexplored, leaving a wide field of regulatory mechanisms yet to be investigated. According to El Fatimy et al. [54], miR-10b, an oncomiR associated with malignant gliomas, was demonstrated to bind U6 snRNA, a crucial part of the spliceosomal machinery, representing an unexpected crossroads between splicing components and miRNAs. The newly discovered nuclear role for miR-10b implies a new therapeutic avenue for treating GBM [54].

Denichenko et al., discussed the development and application of decoy RNA oligonucleotides (DROs) to specifically inhibit the activity of splicing factors. Two distinct DROs, named SF2i1 and SF2i2, were designed to specifically inhibit the activity of the splicing factor SRSF2, preventing it from interacting with its natural RNA targets [82]. These DROs were created to mimic the natural binding sites of SRSF2 on pre-mRNA, effectively “decoying” the splicing factor away from its usual substrates. Inhibiting SRSF2 using SF2i1 and SF2i2 caused altered splicing patterns of many genes demonstrating their ability to selectively regulate splicing events. The study suggests that using SF2i1 and SF2i2 could be a novel therapeutic strategy to correct aberrant splicing in diseases where SRSF2 is implicated, including certain cancers [82].

As part of Wang et al.’s study, a new antisense oligonucleotide (AON) called AON-Ex726 SSO was designed to target the intronic splicing enhancer (ISE) specifically on the hTERT pre-mRNA [83]. The hTERT gene codes for the catalytic subunits of telomerase, which is an enzyme necessary for the maintenance of telomeres length and is often upregulated in cancerous cells including glioma. In order to disrupt normal splicing process, AON-Ex726 SSO was designed to attach onto ISE inside an intron of pre-mRNA. Due to this interference, exon 6 gets excluded during splicing and results in a non-functioning hTETR protein without telomerase activity. The investigation showed that application of AON-Ex726 SSO reduced telomerase activity in glioma cells which is important for the unlimited capability of these tumor cells to divide. Consequently, treated glial tumors displayed less proliferation rates and more apoptosis rates suggesting that this method might serve as a way forward in treatment for glioma [83].

## Conclusion and perspectives

Glioma remains a lethal cancer with challenging therapeutic issues despite ongoing research carried out. Understanding the involvement of oncogenic isoforms produced by AS, as well as deregulation of the spliceosome machinery, could provide answers to the genesis and development of gliomas.

The modifications in AS events including mutations of splicing factors, altered expression levels of RBPs, aberrant m^6^A methylation, and background ncRNAs regulation may lead to either the presentation of splice variants containing oncogenes or the absence of isoforms that act as tumor suppressors, which can, in turn, lead to a drastic change in the behavior of brain tumor [84].

The therapeutic issue of gliomas is exacerbated by the blood-brain barrier, critical to cross, and the cancer intra-heterogeneity which presents a diversity of cancer cells, holding multiple mutations and epigenetic and post-transcriptional aberrations.

Therapeutic strategies that target AS can target three main pathways: the components of the spliceosome or their regulators, the regulatory transcripts involved in AS, and the oncogenic mRNAs or proteins produced by this process.

Molecules causing the inhibition of certain components of the spliceosome machinery, or other new therapeutic molecules such as antisense-oligonucleotides and siRNA may become a prominent therapeutic option [85]. However, molecules with mentioned in vitro outcomes must be evaluated in vivo and combined with carriers that can circulate in the blood for a sufficient time, then be able to cross the blood-brain barrier and present significant specificity for glioma cancer cells.

In the last decades, many studies have been conducted to investigate the implication of protein arginine methyltransferase 5 (PRMT5) in cancer development. This enzyme catalyzes protein methylation in both normal and pathological conditions. The expression of PRMT5 has been reported to correlate with the malignant grade of glioma [86]. One of the roles of this pleotropic enzyme is to methylate snRNPs, showcasing its implication in splicing. Recently, the inhibition of PRMT5 suppressed the growth of patient-derived GBM stem cells through the disruption of multiple splicing events, notably those affecting cell cycle-related genes [87]. One of the inhibitors of PRMT5 (named LLY-283) has shown to have brain-penetrant ability and was able to significantly prolong mice survival [87]. These findings highlight the importance of considering PRMT5 as a promising target to develop treatments for GBM.

Building on our understanding of how dysregulated signaling contributes to glioma progression, another study focused on AS events in the PTPRZ1 gene, which encodes a receptor-type protein tyrosine phosphatase, involved in cell signaling and highly expressed in glioma [88]. An aberrant AS event gives rise to different isoforms of PTPRZ1 that vary in their extracellular domains. With these splicing varieties being exclusive to the tumor cell and absent in normal tissues, being devoid of them, they may serve as a significant target for immunotherapies like CAR-T cells [89]. Nevertheless, the investigation found that the AS events were very heterogeneous, which suggests that spliced isoforms can vary widely in different parts of the tumor and even over time, hence complicating the adaptation of splicing heterogeneity-derived neoantigens to glioma therapy [88].

A first phase I dose-escalation trial was done in other cancers to test H3B-8800, a small SF3b inhibitor that kills spliceosome-mutant tumor cells potently and selectively. The data from the clinical study were recently disclosed, although no whole or partial answers met the international working group criterion [90].

Future clinical trials related to the development of treatments targeting AS in gliomas are expected to promote the prognosis of glioma and GBM particularly.
